# The Global Burden and Trends of Premenopausal Breast Cancer by Modifiable Lifestyle Factors From 1990 to 2021 With Prediction Until 2039

**DOI:** 10.1155/ghe3/5977465

**Published:** 2026-06-16

**Authors:** Baokun Cao, Zhiqiang Xie, Yazhen Chen, Yanting Zhang, Yuxiang Lin, Yan Wang, Haomin Yang

**Affiliations:** ^1^ Department of Epidemiology and Health Statistics, Fujian Provincial Key Laboratory of Environment Factors and Cancer, School of Public Health, Fujian Medical University, Fuzhou, China, fjmu.edu.cn; ^2^ Department of Clinical Laboratory, Fujian Medical University Union Hospital, Fuzhou, China, fjmu.edu.cn; ^3^ Department of Breast Surgery, Zhangzhou Affiliated Hospital of Fujian Medical University, 363000, Zhangzhou, Fujian, China, zzfh.com; ^4^ Department of Breast Surgery, Fujian Medical University Union Hospital, Fuzhou, 350001, China, fjmu.edu.cn; ^5^ Fujian Key Laboratory of Developmental and Neural Biology, College of Life Sciences, Fujian Normal University, Fuzhou, Fujian, 350117, China, fjnu.edu.cn; ^6^ Department of Medical Epidemiology and Biostatistics, Karolinska Institutet, Stockholm, Sweden, ki.se

**Keywords:** burden of disease, predictions, premenopausal breast cancer, risk factors, trends

## Abstract

**Background:**

Although breast cancer incidence has continued to rise worldwide, the disease burden of premenopausal breast cancer remains unclear, particularly given that attributable risk factors may vary across age groups and socioeconomic contexts.

**Methods:**

Regional data on premenopausal breast cancer burden and related risk factors were derived from the updated Global Burden of Disease dataset for 1990–2021, with joinpoint regression applied to characterize time‐dependent changes in disease burden by attributable factor. Disease burden up to 2039 was forecasted using a Bayesian age‐period‐cohort model, with further comparisons of risk‐specific attributable burden.

**Results:**

Countries with a high sociodemographic index (SDI) ranked highest for premenopausal breast cancer incidence (51.5 per 100,000 population), whereas countries with medium–low SDI showed the highest mortality and disability‐adjusted life year (DALY) (7.0 per 100,000 population and 359.2 per 100,000 population, respectively). Disease burden has shown an upward trend in recent years, apart from high SDI areas, while this increase is expected to persist through 2039. Among the attributable risk factors assessed in the GBD framework, diet high in red meat accounted for the greatest share of attributable burden (11.2%), and the mortality and DALY attributed to red meat have both increased, with the largest increase occurring in low–middle SDI countries.

**Conclusions:**

The global burden of premenopausal breast cancer is increasing, with diet high in red meat contributing the greatest proportion of attributable burden among the assessed risk factors. Differences in major attributable risk factors across countries with different SDI levels highlight the need for tailored interventions to reduce the increasing burden of premenopausal breast cancer.

## 1. Introduction

Globally, breast cancer is the leading malignancy among women, accounting for 23.8% of new cancer cases and 15.4% of cancer deaths in 2022 [1]. As compared to postmenopausal women, breast cancer detected among premenopausal women is usually at an advanced stage with poorer prognosis [2, 3]. Intensive studies on the disease burden and major contributors of premenopausal breast cancer may, therefore, provide evidence for precision approaches to disease prevention and targeted intervention, and contribute to the effective allocation of medical resources.

Previous research has evaluated and projected the overall global burden of breast cancer [4], yet relatively limited attention has been paid to premenopausal women [2], despite their distinct risk profiles compared with postmenopausal populations [5, 6]. Some recent studies have investigated the global burden of young breast cancer [7]. However, they failed to incorporate high‐risk premenopausal women aged > 40. Considering the potential changing pattern of risk factors for premenopausal breast cancer in recent years among different countries [8], and the update of the Global Burden of Disease (GBD) dataset including more evidence and data sources [9], a more comprehensive and updated study on the global burden of premenopausal breast cancer is still needed.

Furthermore, several modifiable lifestyle factors, including active and passive smoking [10], regular alcohol consumption [11], and dietary factors [12], have been suggested to influence the likelihood of premenopausal breast cancer. However, the impact of these modifiable lifestyle factors has varied between research evidence from regions at different stages of socioeconomic development, and the burden and trends of premenopausal breast cancer caused by these factors have not been compared.

Therefore, in this study, we aim to characterize the global epidemiology of premenopausal breast cancer (i.e., breast cancer occurring in the female population aged 15–49 years) and show trends and predictions for the burden of disease over time by lifestyle factors. We will also explore attributable risk factors for premenopausal breast cancer across countries disaggregated by sociodemographic index (SDI) and age groups.

## 2. Methods

### 2.1. Study Population

Data were obtained from the 2021 GBD study, which estimates incidence, mortality, and disability‐adjusted life years (DALYs) for 369 diseases and injuries in 204 countries and territories from 1990 to 2021. Data were compiled from structured assessments of population censuses, household‐based surveys, civil registration and vital records, disease registries, disease reporting systems, health service use, atmospheric pollution surveillance, satellite‐based observations, and additional sources [13]. In our study, we focused on female breast cancer diagnosed between 15 and 49 years of age (defined as premenopausal breast cancer). The age at natural menopause shows geographic variation across populations. Although it occurs at around 49 years on average worldwide, women in some regions may experience menopause earlier or later than this average [14, 15]. However, because the net effect would depend on the population distribution and the age‐specific prevalence of menopause, the overall direction of bias could not be determined directly and was therefore further explored in sensitivity analyses. We extracted data on premenopausal breast cancer incidence, mortality, and DALYs in women by age group and country, along with risk factor–attributable mortality and DALYs, from the GBD Results Tool (https://ghdx.healthdata.org/gbd-results-tool).

### 2.2. Definitions

The seven risk factors included in this study were defined according to the GBD 2021 framework, and the attributable burden was estimated relative to the corresponding theoretical minimum risk exposure levels (TMRELs) [16]. Specifically, smoking was defined as current tobacco use or past use with cessation of all tobacco products for at least six months; secondhand smoke referred to exposure to tobacco smoke at home, in the workplace, or in other public settings; high alcohol use was defined as alcohol intake above the region‐, age‐, sex‐, and year‐specific TMREL; high fasting plasma glucose was defined as fasting plasma glucose above the TMREL of 4.8–5.4 mmol/L; low physical activity as physical activity of less than 3600 MET‐minutes per week; diet high in red meat as an intake exceeding the mean level of 0 g (95% UI 0–200) for daily intake of unprocessed red meat; and high body mass index as BMI > 25 kg/m^2^ among individuals aged ≥ 20 years [17–23].

### 2.3. Estimates of the Disease Burden for Premenopausal Breast Cancer

In the GBD 2021, breast cancer is defined as a malignant neoplasm originating from breast epithelial cells and driven by multiple oncogenic factors. Disease burden indicators include incidence, prevalence, mortality, years of life lost (YLL), years lived with disability (YLD), and DALY, where DALY is the combined total of YLL and YLD. DALY is an objective, quantitative methodology that synthesizes the loss of healthy life years due to premature death and disability caused by various diseases. This indicator takes into account various factors, such as mortality, morbidity, severity of the disease, relative importance of age, and discount rate and can objectively reflect the degree of harm caused by the disease to the society and the population [24]. This paper describes the burden of disease using incidence, mortality, and DALY, and describes temporal trends in premenopausal breast cancer disease burden based on annual rates of change in incidence, mortality, and DALY, where incidence, death, and DALYs were age‐standardized to the GBD world population, respectively.

### 2.4. Estimates of Attributable Lifestyle Risk Factors

The GBD 2021 estimates disease burden attributable to 87 individual and combined risk factors at global, regional, and national scales. Attributable disability‐adjusted life year (ADALY) is a DALY‐based metric used to assess the extent to which specific risk factors affect the health of a population. It is based on the idea that if a disease or risk factor can be avoided, then the health of the population will improve, thereby reducing disability, premature death, and the burden of disease. ADALYs are calculated as the product of total DALYs for a specific outcome (e.g., a given disease or injury) and the corresponding population attributable fraction. Attribution scores indicate the degree of association between a particular outcome and a risk factor, i.e., the number of years in which these disorders would have been avoided in the absence of that risk factor [25]. This study examined the trends of ADALY for premenopausal breast cancer by lifestyle risk factors for active smoking, passive smoking, high alcohol use, and diets high in red meat. Attributable DALYs for ages 15–49 were extracted directly using the GBD Results Tool. For subgroups (15–29, 30–39, and 40–49), we aggregated risk‐specific and total DALYs across the constituent 5‐year age cohorts from GBD. Body mass index (BMI) and low physical activity are not included in further analysis considering their inverse association with premenopausal breast cancer [26]. To investigate the effects of these risk factors according to different socioeconomic status, the analyses were stratified by SDI, which is an index of socioeconomic development derived from lagged distributions of income per person, mean educational attainment, and total fertility under 25 years of age, in a formula in which the SDI increases with increases in income and education and decreases in fertility.

### 2.5. Statistical Analysis

In this study, age‐standardized rates and their 95% confidence intervals were estimated through standardization to the GBD 2021 world standard population. We estimated rates per 100,000 population. Temporal patterns in premenopausal breast cancer incidence, DALY, and mortality were assessed by deriving mean annual percentage changes and corresponding 95% confidence intervals using joinpoint regression (JR). The annual point estimates from GBD 2021 were input for the analysis. Temporal patterns in premenopausal breast cancer incidence, mortality, and DALY by SDI from 1990 to 2021 were fitted using JR, where year was the independent variable, and standardized incidence, mortality, and DALY were the dependent variables, with a maximum number of breakpoints of 6, for the analyses. Mortality and DALY for premenopausal breast cancer attributed to the four lifestyle factors were also analyzed using the JR and separated by SDI. We diagnosed the joinpoint model by examining annual residuals (observed–modeled) for zero‐mean and pattern‐free behavior, screening 3*σ* outliers, quantifying errors with root mean square error (RMSE) and symmetric mean absolute percentage error (sMAPE), and evaluating residual autocorrelation.

Global projections of death and DALY attributable to the four lifestyle factors for 2021–2039 were generated with a Bayesian age‐period‐cohort (BAPC) model. The APC model was used in a Bayesian framework and fitted using the INLA package. We modeled counts on a regular year‐by‐age grid using a Poisson log‐link with the logarithm of exposure as an offset. The linear predictor decomposed rates into intercept, age, period, and cohort components, plus a cell‐level (year×age) independent and identically distributed (i.i.d.) error to absorb overdispersion; APC nonidentifiability was handled by sum‐to‐zero constraints. Second‐order random walks (RW2) were applied to smooth the age, period, and cohort effects. Priors were logGamma (1, 5 × 10^−5^) on the RW2 precisions and logGamma (1, 5 × 10^−3^) on the i.i.d. error precision; the intercept had a loose prior. Inference used the deterministic Laplace approximation in R‐INLA. Convergence was declared when the hyperparameter‐mode Hessian was positive definite, and no numerical warnings occurred during fitting. We evaluated model fit and predictive performance using two criteria: the deviance information criterion (DIC, with effective parameters pD) and leave‐one‐out cross‐validation based on the conditional predictive ordinate (CPO), summarized as LOOIC. We assessed calibration via posterior predictive checks (PPC): using INLA marginals to derive 95% posterior predictive intervals (2.5%–97.5%) for each year×age cell and computing the observed coverage. To evaluate the long‐term predictive accuracy and reliability of the BAPC model, data from 1990 to 2016 were used as the training set to forecast the period 2017–2021, and the RMSE, mean absolute error (MAE), and mean absolute percentage error (MAPE) were calculated to assess the model’s goodness of fit and predictive capability. More information on the INLA package is available elsewhere (https://www.r-inla.org/).

## 3. Results

### 3.1. Global Burden and Attributable Risk Factors for Premenopausal Breast Cancer in 2021

**FIGURE 1:**
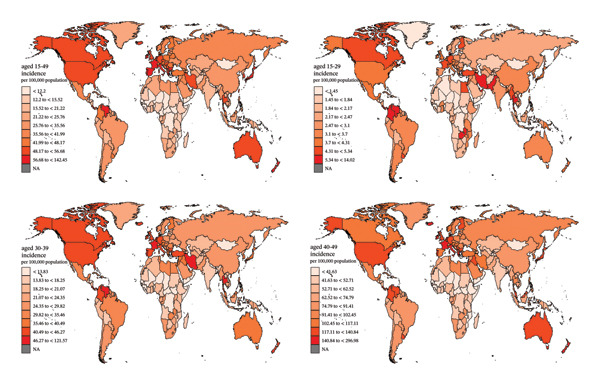
Global distribution of the incidence rate of premenopausal breast cancer by age group, 2021.

In 2021, the global incidence, mortality, and DALY rates for premenopausal breast cancer were 28.8 per 100,000 population (95% CI = 26.8, 30.9), 6.6 per 100,000 population (95% CI = 6.2, 7.1), and 341.7 per 100,000 population (95% CI = 317.7, 366.7), respectively. By SDI category, high SDI countries showed the highest incidence rate, whereas medium–low SDI countries showed the highest mortality and DALY rates for premenopausal breast cancer (Table 1). When examined by country and territory, the highest premenopausal breast cancer rates in 2021 were observed in Western European countries (e.g., Italy, France, and Portugal) and Monaco (Figure 1). Detailed data for the top 10 countries are provided in Table 2.

**TABLE 1:** Indicators for the burden of premenopausal breast cancer by SDI, 1990–2021.

	**Incidence (per 100,000 population)**		**DALYs (per 100,000 population)**		**Mortality rate (per 100,000 population)**	
	**1990**	**2021**	**1990**	**2021**	**1990**	**2021**
Global	19.2 (18.4, 20.2)	28.8 (26.8, 30.9)	298.6 (280.0, 320.0)	341.7 (317.7, 366.7)	5.9 (5.5, 6.3)	6.6 (6.2, 7.1)
High SDI	48.5 (47.5, 49.5)	51.5 (49.8, 53.3)	475.8 (462.1, 491.4)	325.6 (310.0, 343.7)	9.2 (9.1, 9.4)	6.1 (5.9, 6.3)
Medium–high SDI	22.5 (21.2, 24.2)	40.7 (35.8, 46.4)	346.5 (322.4, 375.9)	343.8 (309.1, 385.7)	6.8 (6.4, 7.4)	6.6 (5.9, 7.4)
Medium SDI	11.8 (10.8, 13.1)	29.7 (26.9, 32.7)	243.2 (221.3, 270.2)	356.1 (323.3, 390.6)	4.8 (4.4, 5.3)	7.0 (6.4, 7.7)
Medium–low SDI	8.5 (7.6, 9.5)	18.8 (16.9, 20.7)	225.6 (200.1, 254.5)	359.2 (319.2, 399.6)	4.5 (4.0, 5.0)	7.0 (6.3, 7.8)
Low SDI	7.1 (6.0, 8.5)	11.9 (10.2, 13.8)	217.9 (181.2, 260.7)	288.5 (246.0, 336.3)	4.3 (3.6, 5.1)	5.6 (4.8, 6.5)

Abbreviations: DALYs, disability‐adjusted life years; SDI, sociodemographic index.

**TABLE 2:** Top 10 countries by incidence, mortality, and DALY rates of premenopausal breast cancer in 2021.

Rank	Country	Incidence (per 100,000 population)	Country	DALYs (per 100,000 population)	Country	Mortality rate (per 100,000 population)
1	Monaco	142.5 (94.7, 203.8)	Palau	1047.4 (766.1, 1374.6)	Palau	20.8 (15.1, 27.6)
2	Andorra	77.2 (48.3, 114.1)	American Samoa	1003.4 (752.5, 1304.0)	American Samoa	20.2 (15.2, 26.3)
3	Bahamas	73.4 (55.3, 95.3)	Bahamas	971.0 (733.6, 1266.5)	Bahamas	19.2 (14.5, 25.1)
4	Bermuda	73.1 (56.8, 92.0)	Tokelau	913.6 (688.0, 1223.3)	Tokelau	17.8 (13.2, 24.1)
5	Italy	70.0 (62.8, 76.8)	Niue	891.9 (675.5, 1199.7)	Niue	17.4 (12.9, 23.6)
6	Portugal	69.5 (60.7, 79.6)	Monaco	883.9 (589.2, 1278.1)	Fiji	17.2 (12.2, 23.8)
7	France	69.2 (61.0, 78.4)	Fiji	868.2 (617.9, 1205.1)	Nauru	17.1 (9.3, 27.8)
8	Cook Islands	66.3 (45.2, 93.2)	Nauru	865.2 (469.2, 1408.8)	Cook Islands	16.7 (11.4, 23.5)
9	Malta	65.5 (55.9, 76.3)	Cook Islands	845.4 (579.4, 1194.1)	Monaco	16.5 (11.0, 23.3)
10	Barbados	65.0 (48.8, 84.3)	Marshall Islands	811.1 (458.6, 1291.6)	Marshall Islands	16.2 (9.2, 25.7)

Abbreviation: DALYs: disability‐adjusted life years.

The analyses by age groups indicated that women in the age group of 40–49 years had the highest incidence, mortality, and DALY, and the disease burden was lower in the younger age groups (Supporting Table 1A).

**FIGURE 2:**
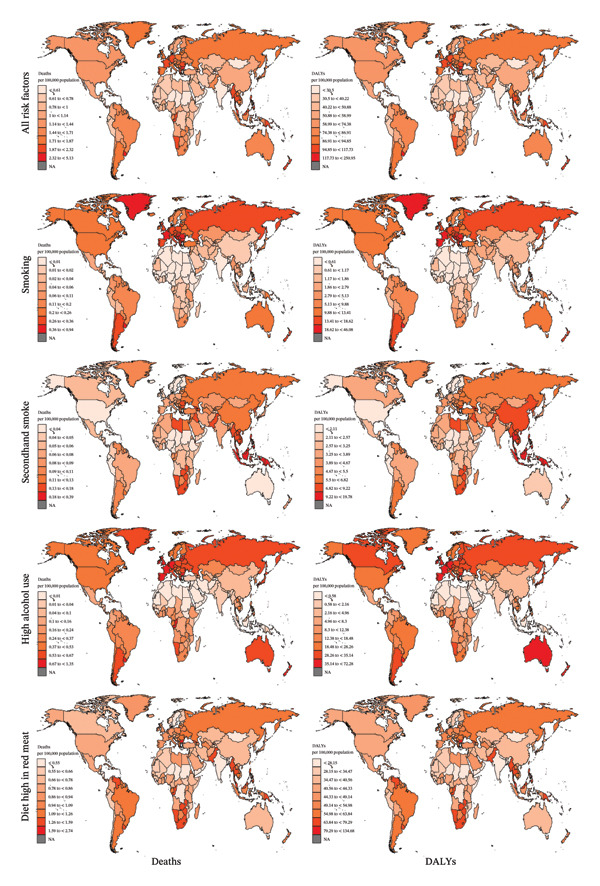
Attributable risk factors for the disease burden of premenopausal breast cancer and their fractions in different regions in 2021. Abbreviations: SDI: sociodemographic index; DALY: disability‐adjusted life years.

Among the risk factors, a diet high in red meat accounted for the greatest share of attributable burden for premenopausal breast cancer, and smoking (1.1%) contributed the least, while high BMI was a protective factor, with attributable deaths and attributable DALYs of −3.8% and −3.7%, respectively (Supporting Table 2 and Supporting Figure 1). Stratified by SDI, high BMI showed the lowest attributable deaths and DALYs in high SDI regions (−5.0%), whereas the highest values were observed in middle SDI regions (−3.2%). DALYs in countries with high SDI, especially in Western Europe, are attributed more to smoking (3.4%), high alcohol use (8.5%), and diets high in red meat content (13.6%), and the proportions attributed to these three factors decreased by SDI (Supporting Table 2 and Supporting Figure 1). Proportion of DALYs attributed to secondhand smoke was highest among the middle–high SDI countries (1.9%), especially in Southeast Asia (Figure 2).

### 3.2. Temporal Trends of Disease Burden for Premenopausal Breast Cancer 1990–2021, Overall and by Attributable Risk Factors

**FIGURE 3:**
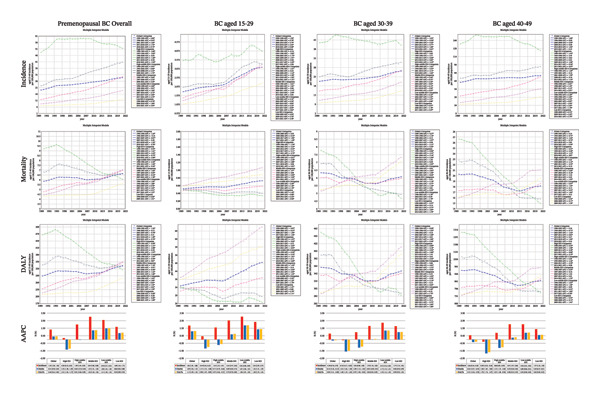
Trends in the disease burden of premenopausal breast cancer in different age groups and different regions from 1990 to 2021. Abbreviations: BC: breast cancer; SDI: sociodemographic index; DALY: disability‐adjusted life years; AAPC: average annual percentage change.

From 1990 to 2021, the premenopausal breast cancer incidence rate increased worldwide (average annual percentage change, AAPC = 1.33% [95% CI 1.30–1.36]), especially in the 15–29 years age group (Figure 3 and Supporting Figure 2). However, there was a large fluctuation for the change in countries with high SDI, with a rapid increase to 1997, subsequently remained stable until 2009, and then a sustained and slow decline to 2021, especially among aged 40–49 years. The fastest increase in premenopausal breast cancer incidence occurred in the medium SDI countries.

Globally, mortality rates and DALYs for premenopausal breast cancer also increased between 1990 and 2021(AAPC = 0.44% [95% CI 0.41–0.46] for DALY and 0.41% [95% CI 0.39–0.43] for mortality, Figure 3). Countries with medium–low SDIs had the highest AAPC for DALY and mortality (Figure 3), whereas the DALY and mortality rates of premenopausal breast cancer declined in countries with high and medium–high SDI, with a downward trend starting from 1995, especially in the 30–39 and 40–49 age groups. For countries with low SDI, the mortality rate and DALY increased continuously across all three age groups. Increased mortality rate and DALY were also observed among middle and low–middle SDI countries for the age group 15–29 (Figure 3).

**FIGURE 4:**
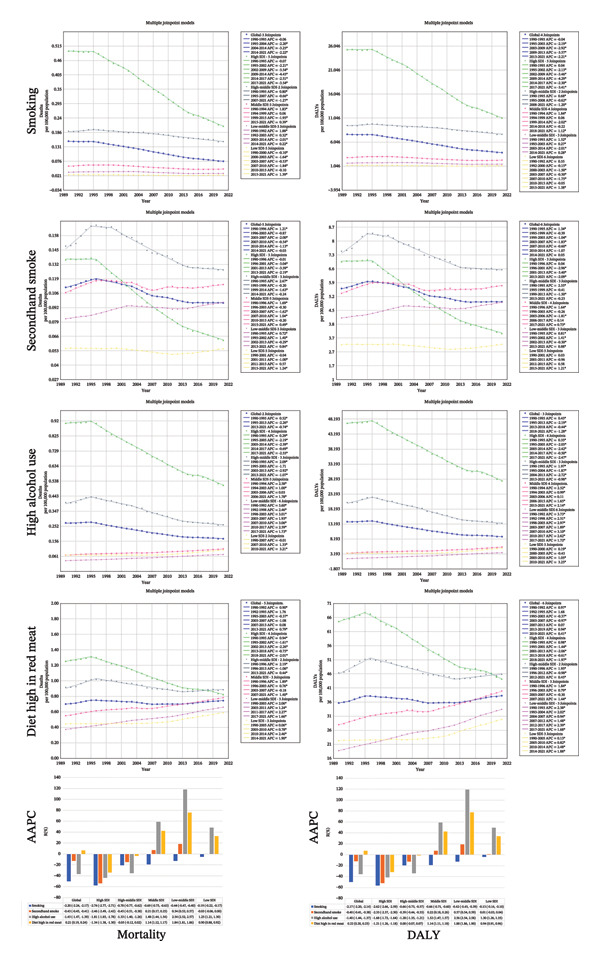
Trends in the disease burden of premenopausal breast cancer by attributable risk factors and different regions from 1990 to 2021. Abbreviations: BC: breast cancer; SDI: sociodemographic index; DALY: disability‐adjusted life years; AAPC: average annual percentage change.

Between 1990 and 2021, both mortality rate and DALY for premenopausal breast cancer attributable to smoking, secondhand smoke, and high alcohol use decreased globally (Figure 4). However, in middle and low–middle SDI countries, mortality rates and DALYs attributed to secondhand smoking and high alcohol use have increased. In low–middle SDI countries, the rates maintained an upward trend throughout 1990 to 2021 for high alcohol use. Interestingly, the global mortality rate and DALYs attributed to high red meat consumption have both increased, with the most substantial rise observed in low–middle SDI countries.

### 3.3. Projections of the Disease Burden Until 2039

**FIGURE 5:**
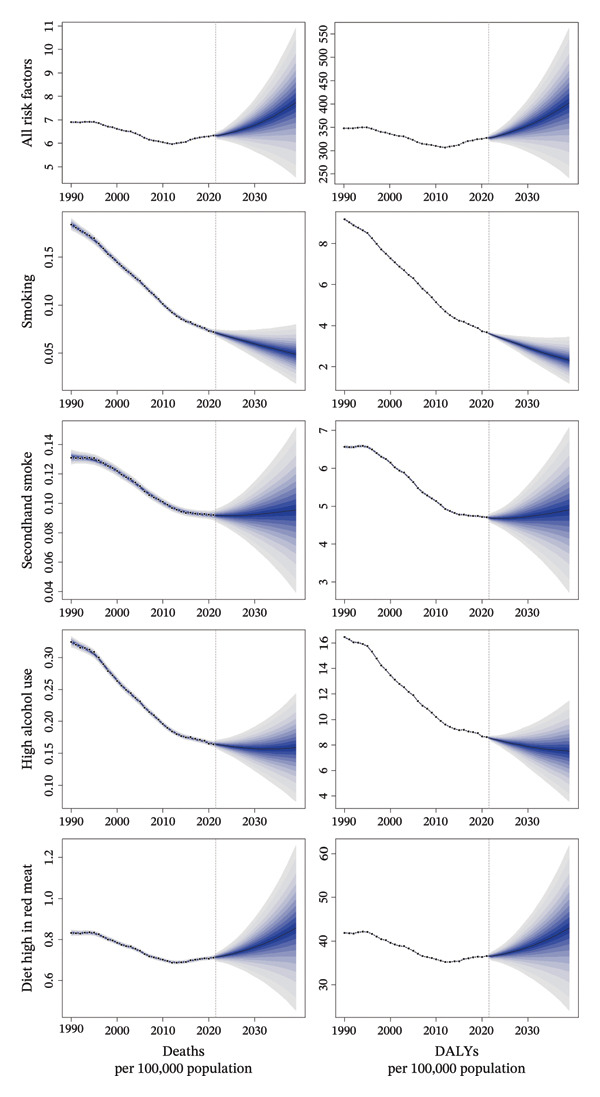
Prediction of global burden of premenopausal breast cancer overall from 2021 – 2039. Abbreviations: BC: breast cancer; DALY: disability‐adjusted life years.

Global mortality and DALY of premenopausal breast cancer are both likely to increase significantly in the 2021–2039 projections (Figure 5). Among the four major attributable risk factors, the mortality rate and DALYs attributed to smoking and high alcohol use are projected to decrease. Conversely, the mortality rate and DALYs due to secondhand smoke and high red meat consumption are expected to increase, with a more pronounced rise associated with high red meat consumption. The BAPC model demonstrated good predictive performance during the validation period (2017–2021), confirming its reliability for long‐term projections up to 2039.

## 4. Discussion

### 4.1. Key Results

Our results showed that premenopausal breast cancer incidence was highest in high SDI countries, while the mortality and DALY were highest in countries with medium and medium–low SDI. In recent years, disease burden has increased in all but high SDI areas, and this rise is expected to continue until 2039. A diet high in red meat contributed the greatest share of attributable burden in all regions, particularly in low–middle SDI countries. Global mortality and DALY from premenopausal breast cancer attributable to high red meat diets have been increasing in recent years, and this upward trend is likely to continue until 2039.

Our analysis showed that countries with the highest incidence rate for premenopausal breast cancer were those with high SDI, which is consistent with studies on the incidence of breast cancer overall [2]. However, their DALYs and mortality rates decreased during the study period and are now lower than those in the rest of the SDI types, except for the low SDI countries. This might be a result of the well‐developed screening programs and medical care in high SDI countries, where more cases of premenopausal breast cancer can be diagnosed early, and patients are better treated and have a better prognosis, which significantly reduces the DALY and mortality rate [27]. However, countries with lower SDI did not benefit from the development of the advanced medical products related to breast cancer prevention and treatment. In addition, differences in registry completeness, coding practices, and access, as well as the lack of stage distribution data in the GBD database, may also affect the observed mortality and DALY patterns.

In our study, a diet high in red meat was an important risk factor for premenopausal breast cancer, corroborating findings from previous research [28]. This may be attributed to heterocyclic amines, which form when meat is cooked at high temperatures and exhibit estrogenic properties in vitro [29, 30]. Furthermore, specific mechanisms by which these chemicals induce carcinogenesis in breast tissue have been suggested [29, 31]. Our study additionally revealed that the impact of a diet high in red meat on premenopausal breast cancer was greater in countries with high SDI compared to those with lower SDI. This may be due to the fact that with economic advancement, countries with high SDI are more likely to adopt Western dietary habits, including diets high in red meat. A study has indicated a positive association between Western‐style dietary patterns and breast cancer risk [32]. However, we also observed increasing mortality and DALY attributed to red meat among low–middle SDI countries, suggesting that controlling diets high in red meat in these countries could represent a crucial preventive strategy for premenopausal breast cancer.

High alcohol use was an important attributable risk factor for premenopausal breast cancer in our study, which is confirmed by previous studies [11]. Our study further found that the contribution of high alcohol use to premenopausal breast cancer was higher in countries with high SDI than those with lower SDI. This may be due to the different rates of high alcohol use in countries with different SDI. One study showed that high SDI countries had the highest rates of high alcohol use, with 72% of women being current drinkers, while medium–low SDI countries had the lowest rates of high alcohol use, with only 8.9% of women being current drinkers [33]. A study suggests a dose–response association between alcohol consumption and breast cancer risk [34]. Therefore, alcohol control in high SDI countries might be an important preventive approach for premenopausal breast cancer.

Both active smoking and secondhand smoking were important attributable factors for premenopausal breast cancer in this study. Previous studies in countries with different SDI showed an early onset of breast cancer and increased risk of premenopausal breast cancer among women exposed with cigarette smoking [35, 36]. In addition, among the five types of SDI classification, the contribution of active smoking to premenopausal breast cancer was highest in high SDI countries and lowest in low SDI countries, which may be due to the fact that, in the female population, the prevalence of smoking was highest in high SDI countries, and the prevalence of active smoking was lowest in low SDI countries [37]. In contrast, the contribution of secondhand smoking to premenopausal breast cancer is highest in middle–high SDI countries, which may be due to a large proportion of women exposed to secondhand smoking in these countries.

Previous evidence indicates that high BMI increases the risk of postmenopausal breast cancer, whereas among premenopausal women, high BMI is linked to a lower risk [6]. The negative relationship between BMI and premenopausal breast cancer is biologically plausible, given that lower BMI may influence growth factors and breast tissue composition in early adulthood [38]. However, given that being overweight increases the risk of many diseases, it is not appropriate to suggest intervention on body weight in order to prevent premenopausal breast cancer.

This study predicted a sustained increase in the disease burden of premenopausal breast cancers, which is similar to the future trend of breast cancer overall [39]. We also found that the largest observed and projected increase in disease burden was associated with high red meat consumption, suggesting that more focus should be placed on this risk factor for breast cancer intervention in this group of premenopausal women. Although we excluded BMI from our disease burden projections, given that it has a negative correlation with premenopausal breast cancer risk, the global rise in BMI remains a major public health challenge. Obesity significantly impacts multiple chronic diseases [40]. Notably, it remains the primary contributor to the burden of postmenopausal breast cancer [41].

### 4.2. Strength and Limitations

Our study comprehensively estimated the burden of disease and trends in premenopausal breast cancer across global, regional, and national settings using multiple indicators, including incidence, DALY, mortality, and attributable risk factors. However, some limitations should be considered when interpreting our findings. First, the quality of data sources varied across countries, which may have influenced our results. For example, limited medical information on death certificates may result in underreporting of premenopausal breast cancer and introduce uncertainty into DALY and mortality estimates. Second, smoking and high alcohol use were positively associated with levels of certain sex hormones (androstenedione, dehydroepiandrosterone sulfate, testosterone, and free testosterone) in premenopausal women, while the contributions attributed to the alteration of serum concentrations of hormones still require further studies in the future. Third, because GBD lacks individual‐level menopause data, we used an age‐based proxy commonly applied in international studies, which might cause misclassifications. In addition, risk factors with negative attributable fractions were not incorporated into the projection models. Although these estimates were retained in the descriptive analyses, their interpretation in future projections remains uncertain. Although regional variations exist, studies have shown that the age at menopause exhibits moderate consistency across geographic regions and ethnic groups, typically occurring around 49 years of age [14]. However, sensitivity analyses using a narrower age range of 15–44 years yielded broadly similar results to the main analysis based on ages 15–49 years, supporting the robustness of the main findings (Supporting Figure 3).

## 5. Conclusion

Premenopausal breast cancer represents an increasing health concern for women, particularly in countries with medium and medium–low SDI. Diet high in red meat is a major contributor to the global burden of premenopausal breast cancer. The variation in attributable risk factors for premenopausal breast cancer across countries with different SDI levels highlights the need for tailored interventions to mitigate its increasing global burden.

## Author Contributions

Baokun Cao and Yazhen Chen had full access to all data included in the study and confirmed both the completeness of the data and the validity of the analytical results. Study concept and design: Haomin Yang and Yan Wang. Data retrieval, statistical analysis, and result interpretation: Baokun Cao, Yazhen Chen, Yanting Zhang, and Yuxiang Lin. Drafting of the manuscript: Baokun Cao and Zhiqiang Xie. Critical revision of the manuscript for important intellectual content: all authors. Statistical analysis: Baokun Cao and Yazhen Chen.

## Funding

Haomin Yang is supported by the Fund for High‐level Talents of the Fujian Medical University [grant no: ZLJH2026001].

## Disclosure

All authors approved the final submitted version. The study sponsor was not involved in designing the study, obtaining or analyzing the data, interpreting the findings, or preparing the manuscript.

## Ethics Statement

This study used only open‐access, summary‐level data and involved no identifiable personal information; therefore, ethical review and informed consent were not applicable.

## Consent

The authors have nothing to report.

## Conflicts of Interest

The authors declare no conflicts of interest.

## Supporting Information

Additional supporting information can be found online in the Supporting Information section.

## Supporting information


**Supporting Information** Supporting 1. Supporting Table 1. Indicators for burden of premenopausal breast cancer in 2021 by age groups. Supporting 2. Supporting Figure 1 Attributable risk factors for premenopausal breast cancer and their fractions in different SDI regions in 2021. Additional supporting information is provided in the supporting materials. Supporting Table 1 presents indicators for the burden of premenopausal breast cancer in 2021 by age groups. Supporting Table 2 presents the population attributable fractions of breast cancer mortality and DALYs for attributable risk factors across SDI levels in 2021. Supporting Figure 1 shows the attributable risk factors for premenopausal breast cancer and their fractions in different age groups in 2021. Supporting Figure 2 presents the AAPC for premenopausal breast cancer incidence by age groups from 1990 to 2021. Supporting Figure 3 shows the sensitivity analysis of incidence, mortality, and DALY trends in women aged 15–44 years versus 15–49 years from 1990 to 2021.

## Data Availability

Breast cancer burden estimates covering 1990–2021 were retrieved from the open‐access Global Health Data Exchange (GHDx) results tool, available at http://ghdx.healthdata.org/gbd-results-tool.
