# Supplementary material for: The Global Burden and Trends of Premenopausal Breast Cancer by Modifiable Lifestyle Factors From 1990 to 2021 With Prediction Until 2039
Source: Glob Health Epidemiol Genom. 2026 Jun 16;2026:5977465. doi: 10.1155/ghe3/5977465 (PMC13270342; doi:10.1155/ghe3/5977465)
Supplement: Supplementary file 1 — Supporting Information Supporting 1. Supporting Table 1. Indicators for burden of premenopausal breast cancer in 2021 by age groups. Supporting 2. Supporting Figure 1 Attributable risk factors for premenopausal breast cancer and their fractions in different SDI regions in 2021. Additional supporting information is provided in the supporting materials. Supporting Table 1 presents indicators for the burden of premenopausal breast cancer in 2021 by age groups. Supporting Table 2 presents the population attributable fractions of breast cancer mortality and DALYs for attributable risk factors across SDI levels in 2021. Supporting Figure 1 shows the attributable risk factors for premenopausal breast cancer and their fractions in different age groups in 2021. Supporting Figure 2 presents the AAPC for premenopausal breast cancer incidence by age groups from 1990 to 2021. Supporting Figure 3 shows the sensitivity analysis of incidence, mortality, and DALY trends in women aged 15–44 years versus 15–49 years from 1990 to 2021. [file GHE3-2026-5977465-s001.docx]

**ONLINE SUPPLEMENTARY**

**Supplementary Table 1**. Indicators for burden of premenopausal breast cancer in 2021 by age groups

**Supplementary Table 2**. Population attributable fractions of breast cancer mortality and DALYs for attributable risk factors across SDI levels in 2021

**Supplementary Figure 1** Attributable risk factors for premenopausal breast cancer and their fractions in different age groups in 2021

**Supplementary Figure 2** AAPC for premenopausal breast cancer incidence by age groups, 1990-2021

**Supplementary Figure 3** Sensitivity analysis of incidence, mortality, and DALY trends in women aged 15–44 years versus 15–49 years, 1990–2021

Supplementary Table 1. Indicators for burden of premenopausal breast cancer in 2021 by age groups

|  | Incidence (per 100,000 population) | | |  | DALYs (per 100,000 population) | | |  | Deaths (per 100,000 population) | | |
| --- | --- | --- | --- | --- | --- | --- | --- | --- | --- | --- | --- |
|  | Age 15-29 | Age 30-39 | Age 40-49 |  | Age 15-29 | Age 30-39 | Age 40-49 |  | Age 15-29 | Age 30-39 | Age 40-49 |
| Global | 3.2(3.1,3.2) | 26.1(26.0,26.2) | 79.1(78.9,79.4) |  | 49.5(49.4,49.7) | 349.2(348.8,349.7) | 869.3(868.5,870.2) |  | 0.7(0.7,0.7) | 6.1(6.0,6.1) | 18.2(18.0,18.3) |
| High SDI | 4.1(3.9,4.2) | 40.2(39.8,40.7) | 125.5(124.7,126.3) |  | 24.9(24.6,25.2) | 284.0(282.8,285.2) | 762.9(760.9,764.9) |  | 0.3(0.3,0.4) | 4.7(4.5,4.8) | 15.0(14.8,15.3) |
| Medium-high SDI | 3.4(3.2,3.5) | 30.8(30.5,31.2) | 95.2(94.6,95.9) |  | 28.4(28.1,28.7) | 289.9(288.9,291.0) | 773.2(771.5,775.0) |  | 0.4(0.4,0.4) | 4.9(4.8,5.1) | 15.8(15.6,16.1) |
| Medium SDI | 3.2(3.1,3.2) | 26.4(26.2,26.7) | 76.6(76.2,77.1) |  | 39.9(39.7,40.2) | 343.4(342.5,344.2) | 885.1(883.7,886.6) |  | 0.6(0.6,0.6) | 6.0(5.9,6.1) | 18.6(18.4,18.8) |
| Medium-low SDI | 3.3(3.2,3.4) | 20.0(19.8,20.3) | 54.5(54.1,55.0) |  | 71.4(71.1,71.7) | 412.8(411.7,413.9) | 983.9(982.1,985.8) |  | 1.1(1.0,1.1) | 7.3(7.2,7.5) | 21.0(20.8,21.3) |
| Low SDI | 2.3(2.2,2.3) | 15.2(14.9,15.5) | 39.8(39.2,40.4) |  | 59.3(58.9,59.7) | 393.7(392.3,395.2) | 908.1(905.4,910.8) |  | 0.9(0.8,0.9) | 7.0(6.8,7.2) | 19.5(19.1,19.9) |

Supplementary Table 2. Population attributable fractions of breast cancer mortality and DALYs for attributable risk factors across SDI levels in 2021

|  | Attributable risk factors for Mortality rate | | | | | |  | Attributable risk factors for DALYs | | | | | |
| --- | --- | --- | --- | --- | --- | --- | --- | --- | --- | --- | --- | --- | --- |
|  | Global | High SDI | High-middle SDI | Middle SDI | Low-middle SDI | Low SDI |  | Global | High SDI | High-middle SDI | Middle SDI | Low-middle SDI | Low SDI |
| Smoking | 1.1 (0.9, 1.4) | 3.5 (2.7, 4.3) | 2.3 (1.7, 2.8) | 0.7 (0.5, 0.8) | 0.4 (0.3, 0.6) | 0.4 (0.3, 0.5) |  | 1.1 (0.9, 1.4) | 3.4 (2.6, 4.3) | 2.2 (1.7, 2.8) | 0.6 (0.5, 0.8) | 0.4 (0.3, 0.5) | 0.4 (0.3, 0.5) |
| Secondhand smoke | 1.5 (-0.4, 3.3) | 1.0 (-0.2, 2.3) | 1.9 (-0.5, 4.3) | 1.6 (-0.4, 3.6) | 1.4 (-0.3, 3.1) | 1.0 (-0.2, 2.2) |  | 1.4 (-0.4, 3.3) | 1.0 (-0.2, 2.3) | 1.9 (-0.5, 4.2) | 1.6 (-0.4, 3.6) | 1.4 (0.3, 3.1) | 1.0 (-2.0, 2.2) |
| High alcohol use | 2.6 (1.8,3.4) | 8.4 (6.1, 10.8) | 3.9 (2.7, 5.3) | 1.6 (1.1, 2.1) | 1.0 (0.7, 1.4) | 1.8 (0.9, 2.6) |  | 2.6 (1.9, 3.5) | 8.5 (6.2, 10.8) | 3.9 (2.7, 5.3) | 1.6 (1.1, 2.1) | 1.0 (0.7, 1.4) | 1.8 (0.9, 2.5) |
| High fasting plasma glucose | 2.4 (-0.7, 5.5) | 2.7 (-0.8, 6.0) | 2.5 (-0.7, 5.9) | 2.5 (-0.7, 5.7) | 2.4 (-0.7, 5.4) | 1.8 (-0.5, 4.2) |  | 2.3 (-0.7, 5.4) | 2.6 (-0.8, 5.9) | 2.4 (-0.7, 5.7) | 2.4 (-0.7, 5.6) | 2.3 (-0.7, 5.2) | 1.7 (-0.5, 4.0) |
| High body-mass index | -3.8 (0.0, -8.4) | -5.0 (-11.1, 0.0) | -3.6 (-8.1, 0.0) | -3.2 (-7.2, 0.0) | -4.1 (-8.9, 0.0) | -3.8 (-8.3, 0.0) |  | -3.7 (-8.4, 0.0) | -5.0 (-11.1, 0.0) | -3.5 (-8.0, 0.0) | -3.2 (-7.2, 0.0) | -4.0 (-8.8, 0.0) | -3.8 (-8.1, 0.0) |
| Diet high in red meat | 11.2 (0.0, 23.8) | 13.6 (0.0, 28.8) | 13.3 (0.0, 28.3) | 11.2 (0.0, 24.0) | 9.4 (0.0, 20.1) | 10.5 (0.0, 22.1) |  | 11.2 (0.0, 23.8) | 13.6 (0.0, 28.7) | 13.3 (0.0, 28.2) | 11.2 (0.0, 24.0) | 9.3 (0.0, 19.8) | 10.4 (0.0, 21.8) |
| Low physical activity | 1.3 (0.3, 2.4) | 1.9 (0.4, 3.4) | 1.3 (0.2, 2.4) | 1.5 (0.3, 2.7) | 1.1 (0.2, 2.0) | 0.9 (0.2, 1.7) |  | 1.3 (0.3, 2.3) | 1.8 (0.4, 3.3) | 1.3 (0.2, 2.3) | 1.5 (0.3, 2.6) | 1.1 (0.2, 2.0) | 0.9 (0.2, 1.6) |


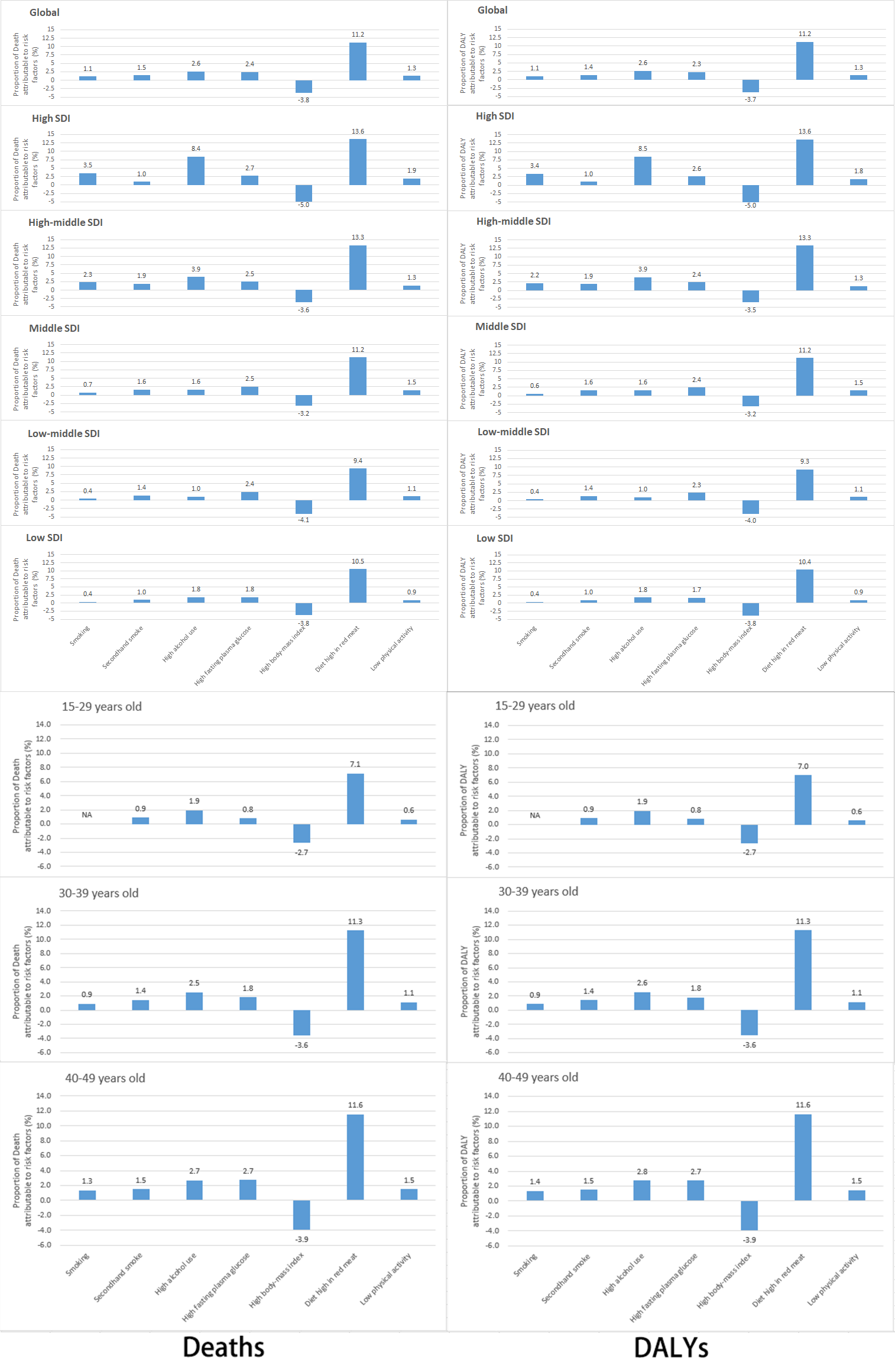


Supplementary Figure 1 Attributable risk factors for premenopausal breast cancer and their fractions in different age groups in 2021


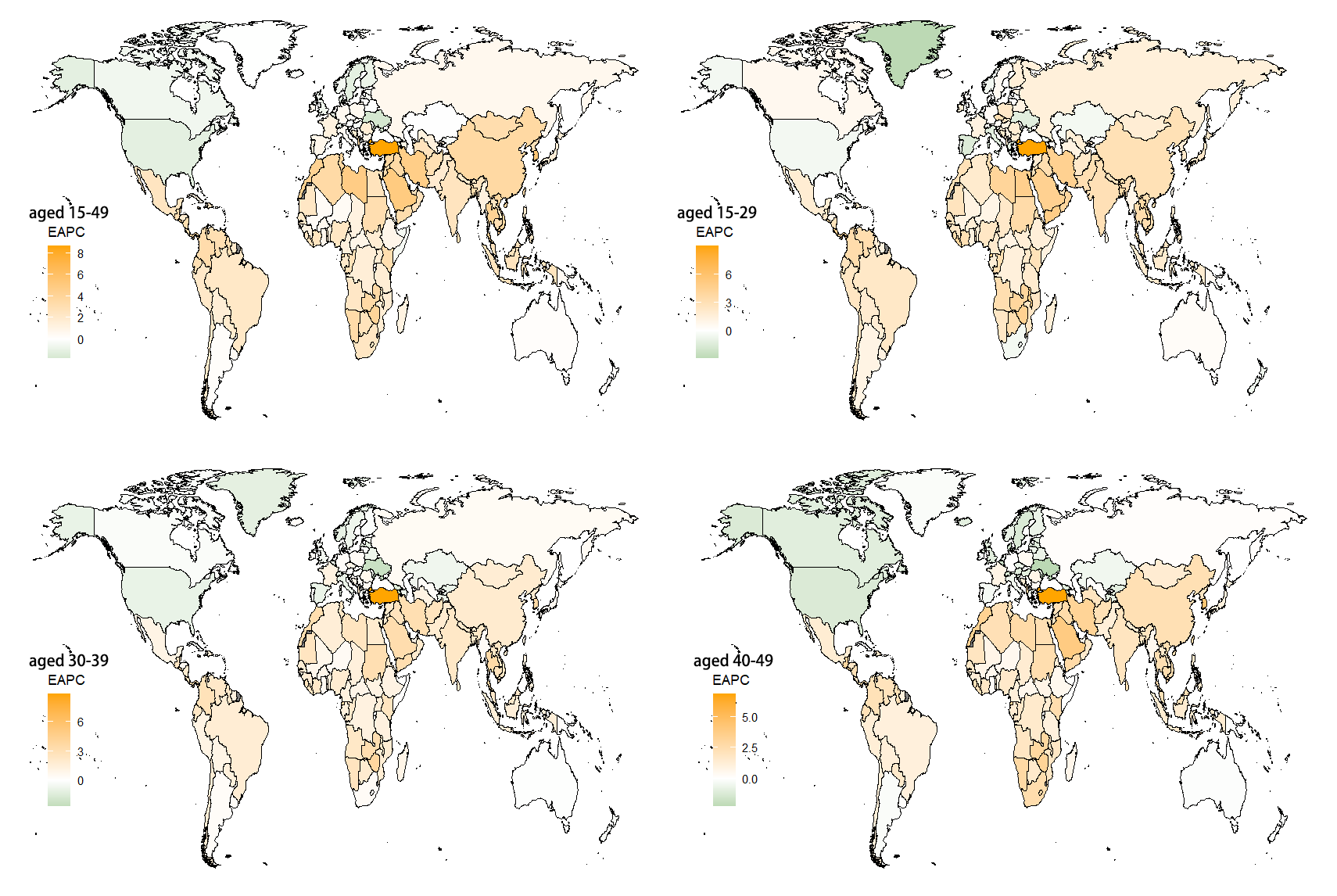
Supplementary Figure 2 AAPC for premenopausal breast cancer incidence by age groups, 1990-2021


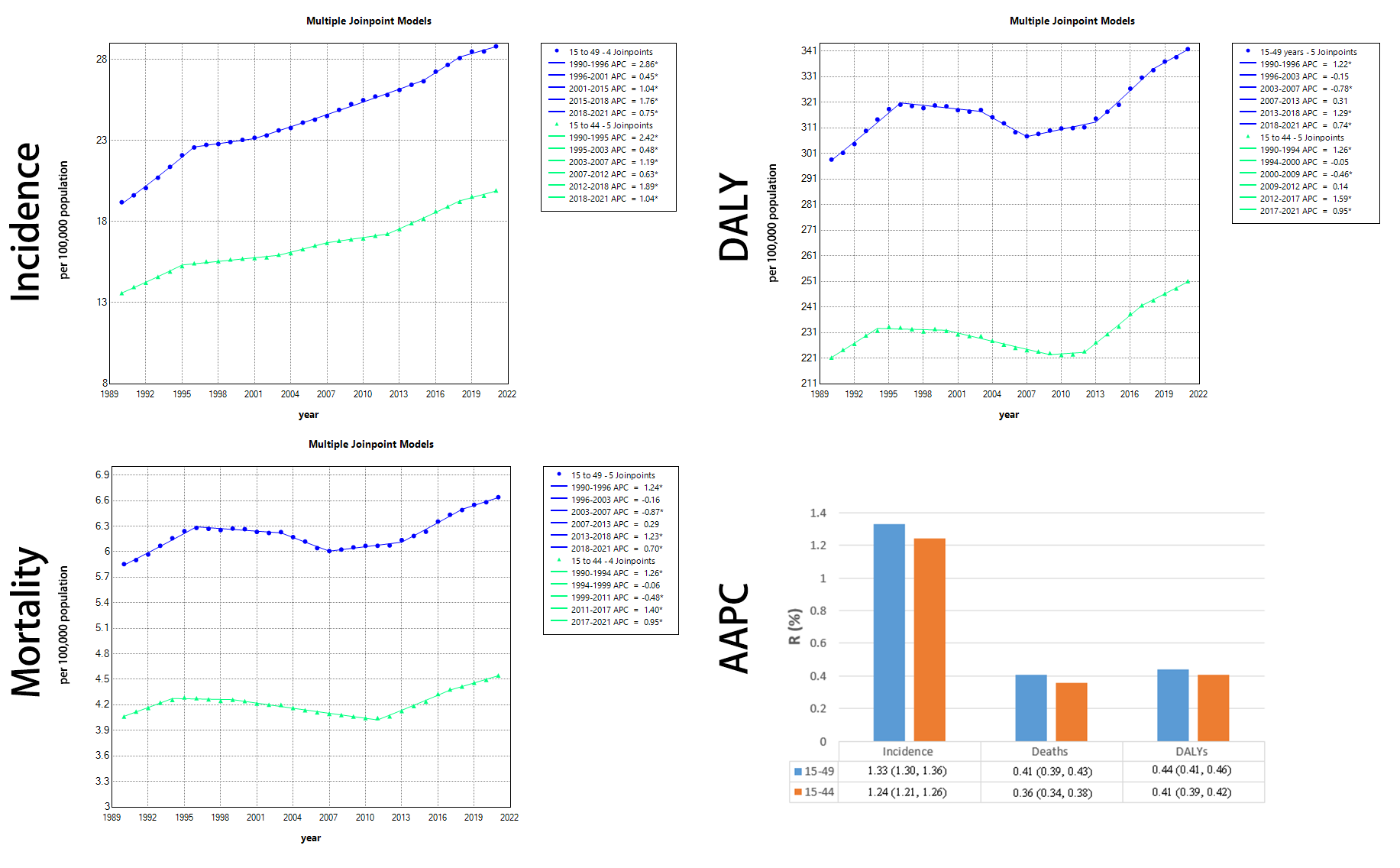


Supplementary Figure 3 Sensitivity analysis of incidence, mortality, and DALY trends in women aged 15–44 years versus 15–49 years, 1990–2021
